# Supporting everyday cognition: Home Technology Tours with people living with dementia

**DOI:** 10.1002/alz.092183

**Published:** 2025-01-09

**Authors:** Arlene Astell, Hira Awan, Danielle Pearson

**Affiliations:** ^1^ Northumbria University, Newcastle upon Tyne United Kingdom; ^2^ University of Reading, Reading United Kingdom; ^3^ University of Toronto, Toronto Canada; ^4^ University Health Network, Toronto Canada

## Abstract

**Background:**

Current off‐the‐shelf technologies contain functionality which can support everyday cognition, such as storing telephone numbers and calendar reminders. These functions can benefit everyone, including people living with dementia. However, knowledge is limited about people living with dementia acquiring and using existing technologies and whether or how they are utilizing these functions.

**Method:**

Technology Tours were completed in the homes of 9 individuals living with dementia, followed by semi‐structured interviews. As they went around their homes the individuals were asked to describe what technologies they used in each room, what they used them for, and how they acquired them. The tours and interviews were transcribed and analyzed using content analysis.

**Result:**

Participants living with dementia described multiple technologies that they use in their everyday lives. These include televisions, stoves and mobility aids, alongside tablets, smart phones, and voice assistants (e.g. Alexa). Their reasons for using their devices were (i) maintaining meaningful activities, (ii) staying connected, and (iii) promoting independence, all of which influenced their well‐being. The usability of devices and safety features were also highlighted.

**Conclusion:**

The findings indicate that people living with dementia use a wide range of technologies to support their everyday cognition. They adopt different functions for different aspects of their lives and would like to access more functionality. This is hindered by the complexity of learning to use and incorporate some potentially helpful devices into their daily lives. These findings can help to dispel myths about the interest and ability of people living with dementia to use digital tools, to encourage wider use of readily available functions and devices, and increased development of new cognitive supports.

